# Purcell-Enhanced Single-Photon Generation from CsPbBr_3_ Quantum Dots in In Situ Selected Laguerre–Gaussian Modes

**DOI:** 10.1021/acsnano.5c20369

**Published:** 2026-02-12

**Authors:** Virginia Oddi, Darius Urbonas, Etsuki Kobiyama, Ioannis Georgakilas, Ihor Cherniukh, Kseniia Shcherbak, Chenglian Zhu, Maryna I. Bodnarchuk, Maksym V. Kovalenko, Rainer F. Mahrt, Gabriele Rainò, Thilo Stöferle

**Affiliations:** † 54174 IBM Research Europe − Zurich, Säumerstrasse 4, Rüschlikon 8803, Switzerland; ‡ Department of Chemistry and Applied Biosciences, 27219 ETH Zurich, Vladimir Prelog Weg 1, Zürich 8093, Switzerland; § Laboratory for Thin Films and Photovoltaics, Empa, Ueberlandstrasse 129, Dübendorf 8600, Switzerland

**Keywords:** perovskite, quantum dots, single photon source, open microcavity, Purcell effect, Laguerre−Gaussian modes

## Abstract

Single photons in Laguerre–Gaussian (LG) beams, which carry orbital angular momentum (OAM), could enable more robust and efficient photonic quantum communication and information processing, as well as enhanced sensitivity in quantum metrology and imaging. However, as most implementations are indirect or require additional mode-shaping elements, the direct generation of single photons with OAM has received growing interest. Colloidal lead halide perovskite quantum dots (QDs) have recently emerged as a versatile material that can produce indistinguishable single photons quasi-deterministically at a high rate. Here, we integrate single CsPbBr_3_ QDs into an open Fabry–Perot microcavity with a nanofabricated Gaussian-shaped deformation, demonstrating Purcell-enhanced single-photon generation into individual cavity modes with up to 18.1 ± 0.2 times accelerated decay, down to tens of picoseconds. By in situ tuning of the cavity resonance, we can selectively couple a single QD to different LG modes and observe the spatial patterns of the generated single-photon beams emitted from the cavity. Our findings may guide the development of high-photon-rate sources that directly generate single-photon LG beams for advanced quantum photonic applications.

## Graphical abstract

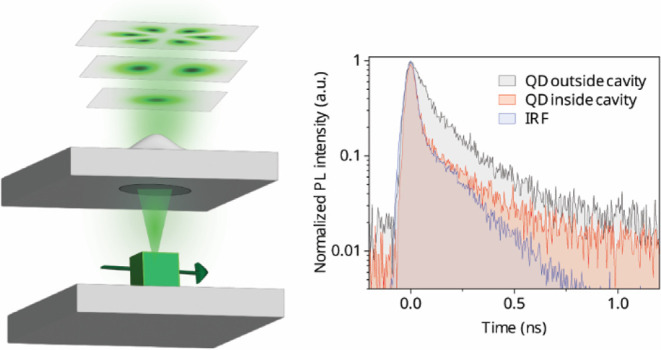

The generation of single photons lies at the heart of quantum technologies. From ultrasecure communication to quantum computing and precision sensing, on demand single-photon sources are essential building blocks. Around the world, researchers are in a race to develop reliable, on-demand single-photon emitters, pushing the boundaries of material science, nanotechnology, and photonic engineering to meet the growing demand of scalable quantum systems.^1–4^ To increase the extraction efficiency and photon quality, single quantum emitters are typically integrated into optical microcavities. The cavity modifies the density of photonic states and thereby, through the Purcell effect, boosts the spontaneous emission rate as described by Fermi’s golden rule. The emission can be funneled into a single, well-defined cavity mode, effectively enhancing brightness, purity and, due to the accelerated decay, also coherence and indistinguishability of the emitted photons.^5,6^ When the emitter is both spectrally and spatially resonant with the cavity mode,^7,8^ the enhancement in emission rate is maximized and quantified by the Purcell factor, *F*_P_ = 3*Q*λ^3^/4π^2^ *n* ^3^ *V*, where *Q* is the quality factor, *V* the effective modal volume, *n* the refractive index, and λ the resonance wavelength of the cavity. Epitaxially grown III–V semiconductor quantum dots (QDs) represent the most exploited solid-state system for quasi-deterministic single photon generation. Successful implementations using epitaxially grown QDs embedded in microcavities exhibit a large Purcell factor^9–11^ of up to several tens and accomplish a high purity, coherence and indistinguishability.^6,12–14^ In a major step toward ideal deterministic sources, tunable, open Fabry–Perot microcavities with a micrometer-sized hemispherical deformation in one of the mirrors, allowing in situ selection of the best QDs, have recently been used to achieve GHz rate generation of high-quality single photons^15^ and system efficiency exceeding 70%.^16^

Colloidal lead halide perovskite QDs with easy and cost-effective solution processability through wet-chemical synthesis,^17^ have attracted increasing attention as single-photon sources that could potentially play a pivotal role in the development of next-generation photonic quantum technologies.^18,19^ In the strong electronic confinement regime, perovskite QDs have achieved single-photon emission purity as high as 98% without spectral filtering.^20^ Weakly confined CsPbBr_3_ QDs at cryogenic temperature, owing to their large oscillator strength, exhibit an extraordinarily fast radiative decay, reaching sub-100 ps lifetime,^21^ about 1 order of magnitude faster than self-assembled III–V semiconductor QDs. Moreover, emission from single CsPbBr_3_ QDs has shown a coherence time of 80 ps (with a radiative lifetime of 210 ps)^22^ and a photon indistinguishability of 56%.^23^ So far, however, only few studies have explored the integration of perovskite QDs into optical microcavities, mostly on ensemble level.^24–27^ Single QDs in a tunable, open microcavity have shown reduced emission line width at room temperature.^28^ However, even at cryogenic temperatures, Purcell factors remained below 2 in circular Bragg grating resonators^29^ and tunable fiber-cavities.^30^

Numerous advanced quantum photonic applications exploit the orbital angular momentum (OAM) of photons, such as realizing high-dimensional entanglement for more efficient and robust quantum communication, encoding qudits^31^ or precision measurements.^32^ Single photons from spontaneous parametric down-conversion or epitaxial QDs are typically transformed into freely adjustable OAM modes, such as Laguerre–Gaussian (LG) beams, employing mode-shaping elements. However, this reduces efficiency and hinders integration. Approaches for direct generation of single photons with OAM using color centers in lithographically defined metasurfaces^33,34^ and epitaxial QDs in ring resonators^35^ have not been able to benefit from the high Purcell enhancement in wavelength-scale microcavities with high *Q*/*V* and do not allow in situ mode selection.

In this work, we integrate single CsPbBr_3_ QDs into a tunable, open Fabry–Perot microcavity with dielectric mirrors comprising a nanofabricated Gaussian-shaped deformation. By comparing the time-resolved emission decay of the same QDs inside and outside of the cavity, we demonstrate a Purcell enhancement of up to 18.1 ± 0.2 at 50 K, accompanied by increased brightness. By using in situ length-tuning of the cavity to bring LG modes of different radial and azimuthal order into resonance with the QD emission, we can coerce the QD to generate single photons directly into these selected LG modes, as evidenced by imaging of the distinct modal patterns.

## Results and Discussion

In our experiments, we use chemically synthesized colloidal CsPbBr_3_ nanocrystals (see Methods) with a size of 25.3 ± 1.5 nm, determined from statistical image analysis of high-resolution transmission electron microscope (HRTEM) images as the one shown in Figure 1a. We chose these relatively large nanocrystals to benefit from their increased emission stability compared to smaller ones, and for their intrinsic fast radiative lifetime. When dispersed in toluene, these QDs exhibit a photoluminescence (PL) quantum yield (QY) of 65% at room temperature. The room-temperature PL emission spectrum, shown in Figure S1, alongside the corresponding absorption spectrum, features a peak energy of 2.39 eV and a full width at half-maximum (fwhm) of 76 meV. When spin-coated onto a substrate and cooled to cryogenic temperatures, the emission red-shifts, and the line width drastically narrows down to sub-meV at 6 K for individual emitters, while the PLQY typically increases near unity.^21^ The fine structure of the bright triplet exciton in single QDs can be clearly resolved, with up to three distinct peaks (Figure 1b) of dominantly linear polarization (inset), depending on the crystal orientation and observation direction.^36,37^ Single-photon emission is established by the characteristic antibunching observed in the second-order correlation function at zero-time delay, *t*, *g* ^(2)^(*t =* 0), as presented in Figure 1c where the uncorrected data are fitted with double-sided exponential functions. The obtained value of *g* ^(2)^(0) *=* 0.13 ± 0.02 confirms single-photon emission, while the relatively high value arises from residual contributions of (charged) multiexciton emission.

**Figure 1:**
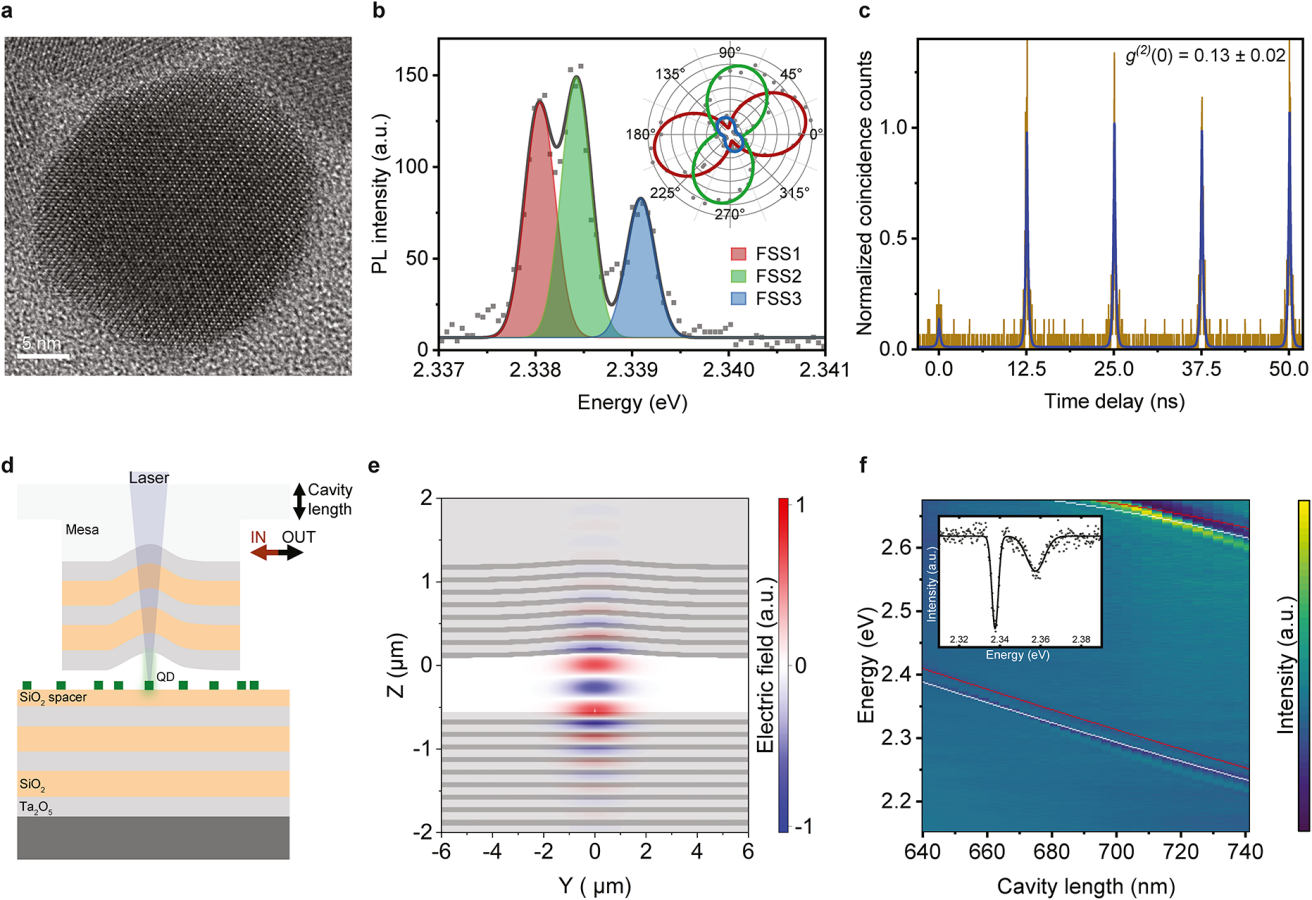
QD emission properties and cavity system. (a) High-resolution transmission electron microscope image of a single 25 nm CsPbBr_3_ QD. (b) PL spectrum of a single CsPbBr_3_ QD at 6 K, fitted with a three-Gaussian-peak function (black solid line) from which the intensity of each fine structure state can be extracted. The intensity of each fine structure line at different angles of a linear polarizer is shown in the inset where the solid lines represent a sin^2^ fit. (c) *g* ^(2)^ measurement, showing a value of *g* ^(2)^(0) = 0.13 ± 0.02. It is retrieved from double-sided exponential fits (blue line) to the raw, uncorrected data (brown line). The normalization was performed by dividing the coincidence counts by the average of the fitted peak values at time delays different from 0. The exciton emission is filtered with a 15 nm-wide tunable bandpass filter. (d) Schematic of the tunable open Fabry–Perot cavity system. (e) Electric field distribution of the LG_00_ mode obtained with 3D FDTD simulation. (f) Spectroscopic white-light reflection measurement of the bare cavity, showing the tuning of LG mode resonances while changing the cavity length. The cavity length is retrieved from transfer-matrix simulations with the two planar modes of different longitudinal order shifted horizontally to account for the Gaussian potential (white points for LG_00_ and red points for LG_01_), see Figure S2c for more details. A maximum *Q*-factor of 623, corresponding to a 3.75 meV fwhm at a peak energy of 2.338 eV, is extracted from a Gaussian fit of the LG_00_ mode (inset).

The single QDs are placed into a tunable Fabry–Perot-type open microcavity (see Methods) with a Gaussian-shaped deformation^38^ to provide tight lateral confinement of the mode, as schematically shown in Figure 1d. The use of two separate, independent mirrors allows not only for length tuning of the cavity but also for easy repositioning of the Gaussian deformation over different QDs as well as for removing the top cavity half to study the same QDs without microcavity for direct comparison. Due to the cylindrical symmetry, this cavity configuration supports Laguerre–Gaussian modes LG_*nl*_, denoted by their radial, *n*, and orbital, *l*, angular momentum quantum numbers.^39,40^ Figure 1e shows a three-dimensional finite-difference time-domain simulation (3D FDTD) of the energetically lowest mode, LG_00_, from which we obtain a quality factor of *Q* = 1941 with a resonant wavelength of λ = 533 nm and an effective modal volume of *V* = 3.3 λ^3^ at an air gap of 705 nm in the cavity center. This configuration results in a calculated Purcell factor of 37.9, see Methods. To investigate the optical properties of the fabricated cavity and the in situ tunability of resonant modes by changing the distance between the cavity halves, we employ spectroscopic white-light reflection measurements (Figure 1f), from which we obtain a *Q*-factor of 623, corresponding to a 3.75 meV fwhm at a peak energy of 2.338 eV (inset). The depth and width of the Gaussian-shaped deformation are chosen such that the different LG modes are spectrally well separated. We operate the cavity in a configuration where the two halves are slightly tilted from perfect parallelism and get in contact far outside of the central region with the Gaussian deformation, drastically reducing the mechanical vibrations and cavity length jitter^41^ while maintaining tunability. Yet, the considerably lower apparent *Q*-factor compared to the theoretical calculation may be due, at least in part, to broadening caused by residual vibrations. As the air gap cannot be measured directly, we perform transfer-matrix simulations of a planar cavity that allow to correlate the observed cavity resonance tuning to the actual cavity length. To account for the Gaussian deformation, which effectively increases the local cavity length, the calculated planar modes are shifted (white points for LG_00_ and red points for LG_01_), as detailed in Figure S2c.

### Purcell-Enhanced Single-Photon Emission

To investigate cavity-enhanced single photon emission in CsPbBr_3_ QDs, we perform measurements at temperatures of 6 K (Figure 2a–c) and 50 K (Figure 2d–f). We use far off-resonant picosecond pulses at 3.06 eV photon energy (see Methods) outside of the mirror spectral stop band (Figure S2a) to excite individual QDs. We show the radiative decay (Figure 2a) and PL spectrum (Figure 2b) of an exemplary QD (QD#1) positioned inside (red line) and outside (gray line) the cavity at 6 K. The characteristic instrument response function (IRF) curve of the single-photon detector is also included in Figure 2a and displayed as blue line. When the QD emission is resonant with the cavity, the emission is funneled into a specific LG mode, LG_00_ (inset of Figure 2b), and an acceleration in the radiative decay, along with an enhancement in the emitted intensity is observed. Extracted lifetimes of three QDs (QD#1, 2, 3) are presented in Figure 2c, with a maximum Purcell factor *F*_P_ = τ_fast,out_/τ_fast,in_ = 4.2 ± 0.1, quantified by fitting the decay traces with a double-exponential function, where τ_fast,in/out_ denotes the time constant of the fast component in- and outside of the cavity. Because in our experimental setup we can directly compare the very same QD inside and outside of the cavity, excited with the same off-resonant excitation power, we can exclude that intensity-dependent processes like biexcitons, trions or Auger quenching would be responsible for the accelerated decay in the cavity. The slow component, τ_slow_, is not Purcell-accelerated as it is likely attributed to delayed refilling from shallow trap states^42^ through tunneling or thermal activation,^43^ or, in some instances, limited by the long tail of the IRF. As shown by the comparison with the IRF trace, a potentially much faster intrinsic acceleration cannot be resolved from this data, as the cavity-accelerated decays at 6 K approach the temporal resolution of the detector.

**Figure 2:**
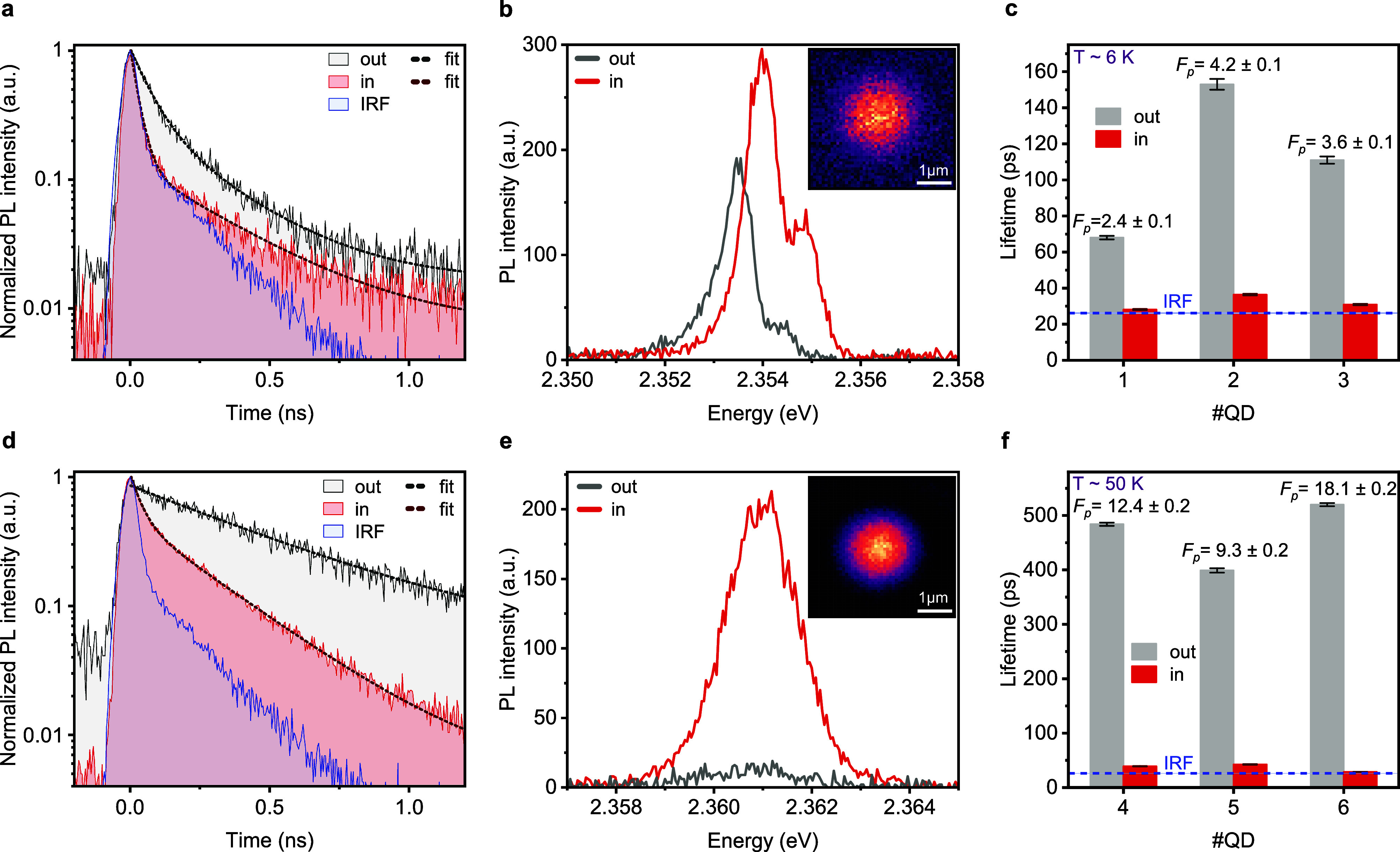
Purcell enhancement at 6 and 50 K. (a), (b) Measurement results at 6 K. Radiative decay (a) and PL spectrum (b) of a QD (#1 in (c)) placed inside (in, red line) and outside (out, gray line) the cavity. The inset in (b) shows the real-space LG_00_ mode profile. (c) Lifetimes and corresponding Purcell factors, *F*_P_, of three QDs at 6 K, extracted from the fast component of a double-exponential fit. The indicated instrument response function (IRF, blue dash line) highlights that the Purcell-enhanced lifetimes are close to the temporal detection limit. A maximum Purcell enhancement of 4.2 ± 0.1 is reached at 6 K. (d), (e) Measurement results at 50 K. Radiative decay (d) and PL spectrum (e) of a QD (#4 in (f)) placed inside (in, red line) and outside (out, gray line) the cavity. The inset in (e) shows the real-space LG_00_ mode profile. (f) Lifetimes and corresponding Purcell factors, *F*_P_, of three QDs at 50 K. Inside the cavity, values are obtained from the fast component of a double-exponential fit, while, outside, from a single-exponential fit. A maximum Purcell enhancement of 18.1 ± 0.2 is reached at 50 K.

This experimental limitation can be circumvented when measuring those QDs at intermediate temperature, as the radiative decay time in perovskite QDs increases with increasing temperature due to reduction of the single-photon superradiance effect,^21^ while the emission line width remains sufficiently narrow to couple well to the cavity mode. Therefore, we present measurements for another exemplary QD (QD#4) at 50 K, showing the radiative decay (Figure 2d) and PL spectrum (Figure 2e), both inside and outside of the cavity, along with the corresponding real space mode profile, LG_00_ (inset of Figure 2e). The relative speed-up in the decay trace is much more pronounced compared to lower temperature, but the fast component remains close to the IRF limit. Additionally, compared to 6 K, the stronger enhancement in the emitted intensity at 50 K may be due to a change in the effective QY, as the Purcell effect can accelerate the radiative decay within the time scale of nonradiative quenching processes. The extracted lifetimes for three QDs (QD#4, 5, 6) are presented in Figure 2f, showing a maximum *F*_P_ = 18.1 ± 0.2. Inside the cavity, the lifetime values are again obtained from a double-exponential fit, while outside of the cavity a single-exponential fit is sufficient, as the QD decay becomes similar or longer than the slower time scale. The emission decay, spectrum and real space mode profile of the additional QDs included in the charts of Figure 2c,f are displayed in Figure S4 and Figure S5, respectively. Additionally, an overview of the extracted parameters τ_fast_, τ_fast,out_/τ_fast,in_, τ_slow_ and the ratio of the spectrally integrated emission intensity inside and outside the cavity, *A*_in_/*A*_out_, for QD#1–6 is provided in Table S1.

The maximum measured Purcell enhancement is lower than the calculated value of 37.9. This may be attributed to imperfections in the cavity fabrication, slightly suboptimal spectral or spatial alignment of the emitter with the optical mode or to the quality of QD emission, as finite line width and a low temporal stability and/or spectral diffusion would reduce the coupling between the QD and the cavity mode. The PL time-series of two presented QDs in Figure S6b and Figure S7 show energy drift over time scales of a few seconds and fluctuations in the emitted intensity.

An additional benefit of Purcell-enhanced coupling of an emitter to a single optical cavity mode stems from the intrinsically provided spectral filtering, reducing the need for (lossy) external filters. This is particularly relevant for CsPbBr_3_ QDs where spectral filtering is important to suppress biexciton emission, which significantly reduces the photon purity^21,22^ due to its high QY in large QDs at low temperature. This is observed in our results as plotted in Figure S6a where we present the *g* ^(2)^(0) of QD#1 outside and inside the cavity. Outside the cavity, when a tunable bandpass filter is used to suppress the biexciton emission, we observe a *g* ^(2)^(0) = 0.25 (pink line) and close to 1 when the filter is removed (gray line). However, in the presence of the cavity, *g* ^(2)^(0) reaches 0.4 even without the use of an additional filter.

### Generation and Control of Photons in LG Modes

Outside of the cavity, the QD emission is observed as a diffraction-limited Gaussian spot, as seen in Figure 3a. In the experiments above, we had tuned the LG_00_ mode into resonance with the QD, also resulting in a Gaussian-shaped cavity emission. However, the cylindrically symmetric cavity also supports higher-order LG modes with nonzero OAM, see schematic in Figure 3b. Hence, when placing a QD inside the cavity and length-tuning the cavity to bring such modes into resonance with the QD, the single photon emission will occur directly into these LG modes. Due to the linearly polarized fine structure lines that make up the excitonic emission of perovskite QDs, both positive and negative OAM quantum numbers can be excited simultaneously; for example, the photons will be in a superposition of left- and right-helical modes. The orientation of the linear dipole is reflected by the phase difference of both states that results in an observed dipole-like pattern in case of LG_01_ instead of the donut mode shape, as illustrated in Figure 3c. Depending on which fine structure line is chosen to overlap best with the cavity resonance, the orientation of the dipole pattern can change because of their different linear polarization.

**Figure 3:**
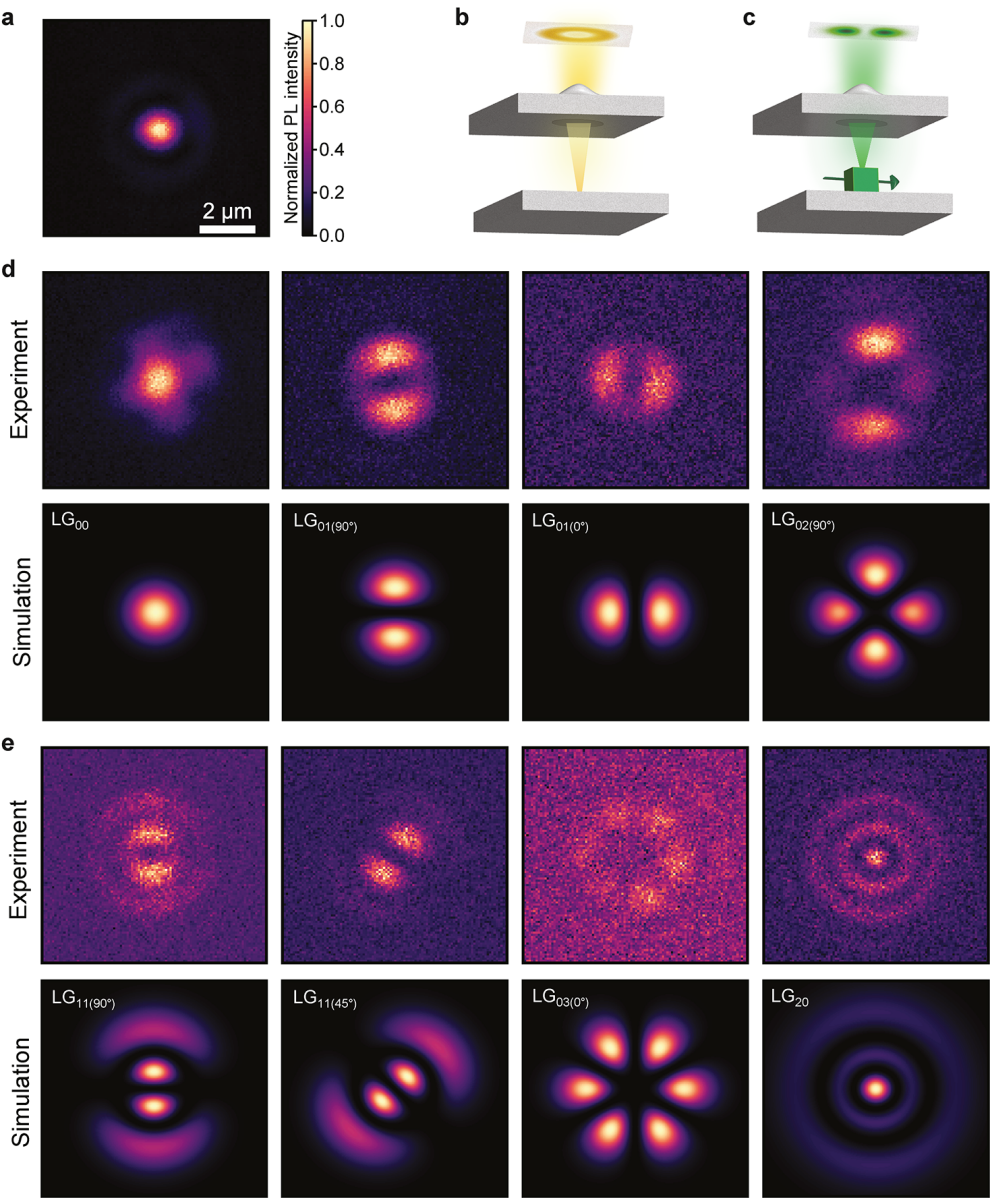
Real-space emission profile of different order Laguerre-Gaussian modes: experiment versus simulation. (a) Real-space emission profile of single QD emission without cavity. (b) Illustration of the cylindrically symmetric LG modes in the cavity, e.g., a donut shape for the case of LG_01_. (c) When a linearly polarized emitter is placed in the cavity, the linear polarization couples to both left- and right-helical photon modes, effectively leading to dipole pattern in the emission of LG_01_. (d), (e) Real-space mode profiles (same length scale as (a)) of different order LG*_nl_* modes: experiment (top row) and simulation (bottom row). In (d), the emission patterns LG_00_, LG_01(90°)_, LG_01(0°)_ and LG_02(90°)_ are obtained from a single QD while in situ tuning the cavity resonance. In (e), LG_11(90°)_ and LG_11(45°)_ were obtained from the same QD (but different QD than (d)) when tuning the cavity, whereas LG_03(0°)_ and LG_20_ come from different QDs each.

The measured emission profiles of different LG_*nl*_ modes resulting from the coupling with single QDs at different cavity lengths are shown in Figure 3(d, e, top row) and compared with the calculated corresponding eigenstates (d, e, bottom row), see Methods for details. In Figure 3d, LG_00_, LG_01(90°)_, LG_01(0°)_ and LG_02(90°)_ are extracted from the same single QD, demonstrating the in situ tunability of our system that enables the selection of a specific LG mode and therefore OAM superposition state. In Figure 3e, LG_11(90°)_ and LG_11(45°)_ originate from the same QD (but different QD than for Figure 3d), whereas LG_03(0°)_ and LG_20_ are obtained from different QDs. Due to the random orientation of the individual QDs and therefore of the fine structure polarization, some QDs couple more efficiently to certain LG modes than others, and slight lateral spatial displacement and/or spectral diffusion of the QDs can also change the coupling efficiency and lead to small discrepancies compared to the simulated mode profiles. The spectrum of each LG_*nl*_ mode is shown in Figure S8.

## Conclusion

In summary, we employed a tunable, open microcavity with an engineered Gaussian-shaped deformation to couple single colloidal CsPbBr_3_ QDs to well-defined LG_*nl*_ modes at 6 and 50 K. We observed Purcell-accelerated emission decays on the order of 30 ps, limited by the temporal resolution of our detector, and a measured Purcell factor of up to 18.1 ± 0.2 at 50 K. We demonstrated the direct generation of single photons into LG modes supporting OAM and that can be controlled by in situ tuning of the cavity resonance. These findings can guide a viable strategy for high-efficiency single-photon sources for quantum applications that utilize the additional dimensions provided by LG states.

## Methods

### QD Synthesis and Basic Characterization

The synthesis of the 25 nm CsPbBr_3_ dots was achieved through the modification of the TOPO-PbBr_2_ approach.^44^ The synthesis was performed by slowly injecting Cs and PbBr_2_ stock solutions into mesitylene. After injection, the solution was treated with a zwitterionic ligand, 2-ammonioethyl (hydroxypolypropylene glycolyl) phosphate (PPG-PEA), which was synthesized according to the procedure described by Morad et al.^45^ The resulting solution was then precipitated with hexane and redispersed in toluene. The synthesis details will be published elsewhere. The HRTEM image shown in Figure 1a was collected using a JEOL JEM-2200FS microscope operated at 200 kV.

For the further sample preparation, CsPbBr_3_ QDs dispersed in solution are diluted up to a factor 10^4^ in toluene starting from a concentration of ∼1 mg/mL. For basic QD characterization shown in Figure 1b,c, the diluted solution is then spin-coated onto a 10 × 10 mm crystalline Si wafer covered with a 3 μm-thick thermal-oxide layer. All samples are prepared in gloveboxes under argon or nitrogen atmosphere.

### Microcavity Fabrication

The dielectric Fabry–Perot microcavity consists of two independent cavity halves. The top part builds on a glass substrate with a mesa, designed to minimize the contact area and sensitivity to particle contamination, allowing to reach gaps between the cavity halves of few hundred nanometers. The mesa structure is fabricated using optical lithography followed by wet etching with concentrated HF, resulting in a mesa that is approximately 30 μm tall and 250 μm wide. A Gaussian-shaped deformation of ∼60 nm depth and ∼4 μm fwhm (Figure S2b) is patterned on top of the mesa using Focused Ion Beam (FIB) milling. Subsequently, 7.5 distributed Bragg reflector (DBR) layer pairs are deposited conformally via Ion Beam Deposition (IBD), composed of alternating quarter-wave layers of SiO_2_ (∼89 nm-thick) and Ta_2_O_5_ (∼62 nm-thick). For the bottom cavity half, 9.5 DBR pairs are deposited on a 20 mm × 20 mm Si substrate via IBD followed by an 85 nm-thick SiO_2_ spacer layer using an e-beam evaporator onto which the diluted QD solution is spin-coated. The DBR stopbands of the top and bottom mirror are shown in Figure S2a.

### Optical Characterization

The two halves of the microcavity are mounted inside a coldfinger liquid-helium-flow cryostat on separate *xyz* nanopositioning stages, with additional tilt stages for the upper half. A home-built μ-PL setup is used for spectroscopic measurements of single QDs and the microcavities. A schematic of the experimental setup is shown in Figure S3. A mode-locked Ti:Sa oscillator (Tsunami, Spectra Physics) with a repetition rate of 80 MHz and pulse duration of 100 fs serves as excitation source after frequency-doubling to 3.06 eV photon energy through a barium borate (BBO) crystal. The light is then guided to the μ-PL setup via an optical single-mode, polarization-maintaining fiber (10 m long, 3 μm core diameter), stretching the pulses in time to several picoseconds. The excitation power is adjusted after the fiber with a graduated neutral-density filter mounted on a motorized linear stage and monitored after a beam splitter (BS) pick-off by a power meter. After passing through a dichroic BS (463 nm edge wavelength, Semrock), the excitation beam is focused on the sample through a microscope objective (Mitutoyo 100X apochromat, NA = 0.7), reaching a 1/*e* ^2^ diameter of 1.9 μm. Typical fluences used to excite single QDs are 0.1–0.85 μJ cm^–2^. The PL emission is collected from the same objective lens and passes through the same dichroic BS and a long-pass filter (450 nm edge wavelength, Semrock). Additionally, a tunable band-pass filter (Semrock) can be inserted as needed. Then, a flip mirror directs the light either to a Quantitative-CMOS camera (ORCA-Quest, Hamamatsu Photonics) or to a 750 mm-long monochromator (Acton) equipped with a grating with 1800 lines/mm and an EMCCD detector (ProEM, Princeton Instruments). For *g* ^(2)^ and lifetime measurements, either a 50/50 BS (for simultaneous acquisition with spectra) or a mirror is placed in front of the monochromator to send the light to a Hanbury Brown-Twiss setup with an unpolarized 50/50 BS and two avalanche photodiodes (PDM, Micro Photon Devices) that are recorded with a time-correlated single-photon counting system (PicoHarp, PicoQuant). For the basic empty cavity reflection characterization in the same setup at room temperature, a fiber-coupled halogen lamp is used as excitation source, which is focused on the sample to a spot diameter of 5.1 μm.

To achieve optimal coupling between the QD and the cavity mode, a systematic alignment procedure is followed. First, the two cavity halves are aligned parallel by tilting the top mirror until the individual reflections of the excitation laser overlap in *k*-space on the camera. Then, the Gaussian deformation is precisely positioned over the QD and brought into focus. Once aligned, the cavity length is gradually reduced by moving the bottom part up until the QD and cavity mode are spectrally in resonance and the emitted intensity in the spectrum is maximized while at the same time the emitted light is monitored in real space with the camera in order to select the desired LG mode. After the measurements with the QD in the cavity, the top part is fully removed from the beam path, allowing the study of the same QD outside the cavity. As can be seen in the work of Zhu et al.,^21^ the decay time for single CsPbBr_3_ QDs of the same size at low temperature can vary significantly, with differences exceeding 100 ps and reaching up to a 2-fold variation at 6 K. Hence, the possibility to make this direct comparison of the same QDs in- and outside of the cavity is important to obtain reliable and precise Purcell factors.

### Photonic Simulations

For the numerical 3D FDTD simulations, we use the commercial software package Lumerical with the refractive indices of the materials experimentally obtained from variable-angle spectroscopic ellipsometry (Wollam VASE). To calculate the effective modal volume *V*, we use the expression *V* = _v_∫*ε*(***r***)| ***E*** (***r***)|^2^ d^3^ ***r***/max­(*ε*(***r***)| ***E*** (***r***)|^2^), where ***E*** (***r***) is the electric field and *ε*(***r***) is the electric permittivity. The theoretical Purcell factor is calculated using $F_{P}=6\pi c^{3}Q/\omega_{c}^{3}V$ , where *c* is the speed of light, *ω_c_* is the angular frequency of the optical mode, and *Q* the quality factor. This definition considers the optimal case in which the dipole is spectrally narrow and resonant with the cavity mode, located at the electric field antinode and aligned with the local electric field.

We use transfer matrix simulations to calculate the reflectance of the multilayer structure as a function of the cavity length, defining a central wavelength of 530 nm for the DBR stopbands. The model assumes normal incidence and accounts for the wavelength-dependent dispersion of the refractive indices of Ta_2_O_5_ and SiO_2_. The cavity length is defined as the thickness of the air gap between the flat DBR mirrors.

In order to calculate the LG modes in the Gaussian deformation, we solve the 2D time-independent Schrödinger equation for the photon as particle in the Gaussian potential well, using the QMsolve Python package. We assume an effective mass of *m*_eff_ *= E*/*c* ^2^, with *E* being the photon energy of 2.32 eV and *c* the speed of light. The confining potential is defined as a Gaussian well with a depth of 94 meV (corresponding to 60 nm actual depth at ∼700 nm cavity length, see caption of Figure S2c) and a fwhm of 4 μm. To implicitly slightly lift the degeneracy of the cylindrically symmetric eigenstates, we introduce a small anisotropy, up to 2%, by modifying the Gaussian’s ellipticity in the calculation. This is used to effectively account for the linear QD dipole orientation, which when coupled to the LG cavity modes leads to photon emission into superposition states of left- and right-handed helicity whose phase relation reflects the azimuthal orientation of the linearly polarized dipole.

## Supplementary Material



## Data Availability

The preprint version of this work is Oddi, V.; Urbonas, D.; Kobiyama, E.; Georgakilas, I.; Cherniukh, I.; Shcherbak, K.; Zhu, C.; Bodnarchuk, M. I.; Kovalenko, M. V.; Mahrt, R. F.; Rainò, G.; Stöferle, T. Purcell-enhanced single-photon generation from CsPbBr_3_ quantum dots in in-situ selected Laguerre-Gaussian modes. 2025, 2510.01837. arXiv. 10.48550/arXiv.2510.01837 (accessed Feb. 02, 2026). The data underlying this study are openly available in Zenodo at 10.5281/zenodo.18466983.

## Notes

PL and absorption spectra of CsPbBr_3_ QDs, additional details on the cavity characterization, and a schematic of the experimental setup; decay traces, PL spectrum, and real-space mode profiles of QD#2, 3, 5, and 6; measurements of *g* ^(2)^(0) and time-series for QD#1, as well as time-series measurements for QD#5; the spectra of the LG_*nl*_ modes (PDF)

## #

Paul Scherrer Institute, Forschungsstrasse 111, 5232 Villigen, Switzerland

## ∥

Institute of Science and Technology Austria, Am Campus 1, 3400 Klosterneuburg, Austria

## References

[1] Aharonovich I., Englund D., Toth M. (2016). Solid-State Single-Photon Emitters. Nat. Photonics.

[2] Thomas S., Senellart P. (2021). The Race for
the Ideal Single-Photon Source Is On. Nat. Nanotechnol..

[3] Couteau C., Barz S., Durt T., Gerrits T., Huwer J., Prevedel R., Rarity J., Shields A., Weihs G. (2023). Applications
of Single Photons to Quantum Communication and Computing. Nat. Rev. Phys..

[4] Fox A. M. (2025). Solid-State
Quantum Emitters. Adv. Quantum Technol..

[5] Lodahl P., Mahmoodian S., Stobbe S. (2015). Interfacing Single Photons and Single
Quantum Dots with Photonic Nanostructures. Rev.
Mod. Phys..

[6] Senellart P., Solomon G., White A. (2017). High-Performance Semiconductor
Quantum-Dot
Single-Photon Sources. Nat. Nanotechnol..

[7] Purcell E. M., Torrey H. C., Pound R. V. (1946). Resonance Absorption by Nuclear Magnetic
Moments in a Solid. Phys. Rev..

[8] Gérard J., Sermage B., Gayral B., Legrand B., Costard E., Thierry-Mieg V. (1998). Enhanced Spontaneous Emission by Quantum Boxes in a
Monolithic Optical Microcavity. Phys. Rev. Lett..

[9] Gerard J.-M., Gayral B. (1999). Strong Purcell Effect for InAs Quantum Boxes in Three-Dimensional
Solid-State Microcavities. J. Light. Technol..

[10] Liu F., Brash A. J., O’Hara J., Martins L. M. P. P., Phillips C. L., Coles R. J., Royall B., Clarke E., Bentham C., Prtljaga N., Itskevich I. E., Wilson L. R., Skolnick M. S., Fox A. M. (2018). High Purcell Factor
Generation of Indistinguishable On-Chip Single Photons. Nat. Nanotechnol..

[11] Rickert L., Vajner D. A., von Helversen M., Schall J., Rodt S., Reitzenstein S., Liu H., Li S., Ni H., Niu Z., Heindel T. (2025). High Purcell
Enhancement in Quantum-Dot Hybrid Circular
Bragg Grating Cavities for GHz Clock Rate Generation of Indistinguishable
Photons. ACS Photonics.

[12] Santori C., Fattal D., Vucković J., Solomon G. S., Yamamoto Y. (2002). Indistinguishable
Photons from a Single-Photon Device. Nature.

[13] Somaschi N., Giesz V., De Santis L., Loredo J. C., Almeida M. P., Hornecker G., Portalupi S. L., Grange T., Antón C., Demory J., Gómez C., Sagnes I., Lanzillotti-Kimura N. D., Lemaítre A., Auffeves A., White A. G., Lanco L., Senellart P. (2016). Near-Optimal Single-Photon Sources in the Solid State. Nat. Photonics.

[14] Wang H., He Y.-M., Chung T.-H., Hu H., Yu Y., Chen S., Ding X., Chen M.-C., Qin J., Yang X., Liu R.-Z., Duan Z.-C., Li J.-P., Gerhardt S., Winkler K., Jurkat J., Wang L.-J., Gregersen N., Huo Y.-H., Dai Q., Yu S., Höfling S., Lu C.-Y., Pan J.-W. (2019). Towards Optimal
Single-Photon Sources from Polarized Microcavities. Nat. Photonics.

[15] Tomm N., Javadi A., Antoniadis N. O., Najer D., Löbl M. C., Korsch A. R., Schott R., Valentin S. R., Wieck A. D., Ludwig A., Warburton R. J. (2021). A Bright
and Fast Source of Coherent
Single Photons. Nat. Nanotechnol..

[16] Ding X., Guo Y.-P., Xu M.-C., Liu R.-Z., Zou G.-Y., Zhao J.-Y., Ge Z.-X., Zhang Q.-H., Liu H.-L., Wang L.-J., Chen M.-C., Wang H., He Y.-M., Huo Y.-H., Lu C.-Y., Pan J.-W. (2025). High-Efficiency
Single-Photon Source above the Loss-Tolerant Threshold for Efficient
Linear Optical Quantum Computing. Nat. Photonics.

[17] Protesescu L., Yakunin S., Bodnarchuk M. I., Krieg F., Caputo R., Hendon C. H., Yang R. X., Walsh A., Kovalenko M. V. (2015). Nanocrystals
of Cesium Lead Halide Perovskites (CsPbX3, X = Cl, Br, and I): Novel
Optoelectronic Materials Showing Bright Emission with Wide Color Gamut. Nano Lett..

[18] Kagan C. R., Bassett L. C., Murray C. B., Thompson S. M. (2021). Colloidal Quantum
Dots as Platforms for Quantum Information Science. Chem. Rev..

[19] Zhu J., Li Y., Lin X., Han Y., Wu K. (2024). Coherent Phenomena
and Dynamics of Lead Halide Perovskite Nanocrystals for Quantum Information
Technologies. Nat. Mater..

[20] Zhu C., Marczak M., Feld L., Boehme S. C., Bernasconi C., Moskalenko A., Cherniukh I., Dirin D., Bodnarchuk M. I., Kovalenko M. V., Rainò G. (2022). Room-Temperature, Highly Pure Single-Photon
Sources from All-Inorganic Lead Halide Perovskite Quantum Dots. Nano Lett..

[21] Zhu C., Boehme S. C., Feld L. G., Moskalenko A., Dirin D. N., Mahrt R. F., Stöferle T., Bodnarchuk M. I., Efros A. L., Sercel P. C., Kovalenko M. V., Rainò G. (2024). Single-Photon Superradiance in Individual Caesium Lead
Halide Quantum Dots. Nature.

[22] Utzat H., Sun W., Kaplan A. E. K., Krieg F., Ginterseder M., Spokoyny B., Klein N. D., Shulenberger K. E., Perkinson C. F., Kovalenko M. V., Bawendi M. G. (2019). Coherent Single-Photon
Emission from Colloidal Lead Halide Perovskite Quantum Dots. Science.

[23] Kaplan A. E. K., Krajewska C. J., Proppe A. H., Sun W., Sverko T., Berkinsky D. B., Utzat H., Bawendi M. G. (2023). Hong–Ou–Mandel
Interference in Colloidal CsPbBr3 Perovskite Nanocrystals. Nat. Photonics.

[24] Yang Z., Pelton M., Bodnarchuk M. I., Kovalenko M. V., Waks E. (2017). Spontaneous Emission Enhancement
of Colloidal Perovskite Nanocrystals
by a Photonic Crystal Cavity. Appl. Phys. Lett..

[25] Fong C. F., Yin Y., Chen Y., Rosser D., Xing J., Majumdar A., Xiong Q. (2019). Silicon Nitride
Nanobeam Enhanced Emission from All-Inorganic Perovskite
Nanocrystals. Opt. Express.

[26] Purkayastha P., Gallagher S., Jiang Y., Lee C.-M., Shen G., Ginger D., Waks E. (2024). Purcell Enhanced Emission and Saturable
Absorption of Cavity-Coupled CsPbBr3 Quantum Dots. ACS Photonics.

[27] Georgakilas I., Tiede D., Urbonas D., Mirek R., Bujalance C., Caliò L., Oddi V., Tao R., Dirin D. N., Rainò G., Boehme S. C., Galisteo-López J. F., Mahrt R. F., Kovalenko M. V., Miguez H., Stöferle T. (2025). Room-Temperature
Cavity Exciton-Polariton Condensation in Perovskite Quantum Dots. Nat. Commun..

[28] Farrow T., Dhawan A. R., Marshall A. R., Ghorbal A., Son W., Snaith H. J., Smith J. M., Taylor R. A. (2023). Ultranarrow Line
Width Room-Temperature Single-Photon Source from Perovskite Quantum
Dot Embedded in Optical Microcavity. Nano Lett..

[29] Jun S., Kim J., Choi M., Kim B. S., Park J., Kim D., Shin B., Cho Y.-H. (2024). Ultrafast and Bright Quantum Emitters
from the Cavity-Coupled Single Perovskite Nanocrystals. ACS Nano.

[30] Said, Z. ; Trouche, M. C. ; Borel, A. ; Amara, M.-R. ; Reichel, J. ; Voisin, C. ; Diederichs, C. ; Chassagneux, Y. Cavity Quantum Electrodynamics with Single Perovskite Quantum Dots. arXiv, 2025. 10.48550/arXiv.2503.20411 41881767

[31] Erhard M., Krenn M., Zeilinger A. (2020). Advances in
High-Dimensional Quantum
Entanglement. Nat. Rev. Phys..

[32] Kam A., Tsesses S., Ilin Y., Cohen K., Lumer Y., Fridman L., Lotan S., Patsyk A., Nemirovsky-Levy L., Orenstein M., Segev M., Bartal G. (2025). Near-Field Photon Entanglement
in Total Angular Momentum. Nature.

[33] Wu C., Kumar S., Kan Y., Komisar D., Wang Z., Bozhevolnyi S. I., Ding F. (2022). Room-Temperature on-Chip Orbital
Angular Momentum Single-Photon Sources. Sci.
Adv..

[34] Liu X., Kan Y., Kumar S., Kulikova L. F., Davydov V. A., Agafonov V. N., Zhao C., Bozhevolnyi S. I. (2024). Ultracompact Single-Photon Sources
of Linearly Polarized Vortex Beams. Adv. Mater..

[35] Chen B., Wei Y., Zhao T., Liu S., Su R., Yao B., Yu Y., Liu J., Wang X. (2021). Bright Solid-State Sources for Single
Photons with Orbital Angular Momentum. Nat.
Nanotechnol..

[36] Becker M. A., Vaxenburg R., Nedelcu G., Sercel P. C., Shabaev A., Mehl M. J., Michopoulos J. G., Lambrakos S. G., Bernstein N., Lyons J. L., Stöferle T., Mahrt R. F., Kovalenko M. V., Norris D. J., Rainò G., Efros A. L. (2018). Bright Triplet Excitons in Caesium Lead Halide Perovskites. Nature.

[37] Oddi V., Zhu C., Becker M. A., Sahin Y., Dirin D. N., Kim T., Mahrt R. F., Even J., Rainò G., Kovalenko M. V., Stöferle T. (2024). Circularly Polarized Luminescence
Without External Magnetic Fields from Individual CsPbBr3 Perovskite
Quantum Dots. ACS Nano.

[38] Ding F., Stöferle T., Mai L., Knoll A., Mahrt R. F. (2013). Vertical
Microcavities with High $Q$ and Strong Lateral Mode Confinement. Phys. Rev. B.

[39] Allen L., Beijersbergen M. W., Spreeuw R. J. C., Woerdman J. P. (1992). Orbital
Angular
Momentum of Light and the Transformation of Laguerre-Gaussian Laser
Modes. Phys. Rev. A.

[40] Plick W. N., Krenn M. (2015). Physical Meaning of
the Radial Index of Laguerre-Gauss Beams. Phys.
Rev. A.

[41] Pallmann M., Eichhorn T., Benedikter J., Casabone B., Hümmer T., Hunger D. (2023). A Highly Stable and Fully Tunable Open Microcavity
Platform at Cryogenic Temperatures. APL Photonics.

[42] Becker M. A., Bernasconi C., Bodnarchuk M. I., Rainò G., Kovalenko M. V., Norris D. J., Mahrt R. F., Stöferle T. (2020). Unraveling
the Origin of the Long Fluorescence Decay Component of Cesium Lead
Halide Perovskite Nanocrystals. ACS Nano.

[43] Efros A. L., Nesbitt D. J. (2016). Origin and Control of Blinking in Quantum Dots. Nat. Nanotechnol..

[44] Akkerman Q. A., Nguyen T. P. T., Boehme S. C., Montanarella F., Dirin D. N., Wechsler P., Beiglböck F., Rainò G., Erni R., Katan C., Even J., Kovalenko M. V. (2022). Controlling the Nucleation and Growth Kinetics of Lead
Halide Perovskite Quantum Dots. Science.

[45] Morad V., Stelmakh A., Svyrydenko M., Feld L. G., Boehme S. C., Aebli M., Affolter J., Kaul C. J., Schrenker N. J., Bals S., Sahin Y., Dirin D. N., Cherniukh I., Raino G., Baumketner A., Kovalenko M. V. (2024). Designer
Phospholipid Capping Ligands for Soft Metal Halide Nanocrystals. Nature.

