# Supplementary material for: Purcell-Enhanced Single-Photon Generation from CsPbBr3 Quantum Dots in In Situ Selected Laguerre–Gaussian Modes
Source: ACS Nano. 2026 Feb 12;20(7):6167–75. doi: 10.1021/acsnano.5c20369 (PMC12947728; doi:10.1021/acsnano.5c20369)
Supplement: Supplementary file 1 [file nn5c20369_si_001.pdf]

Supporting Information

Purcell-Enhanced Single-Photon Generation from $CsPbBr_3$ Quantum Dots in In Situ Selected Laguerre-Gaussian Modes

Virginia Oddi[1,2], Darius Urbonas[1], Etsuki Kobiyama[1], Ioannis Georgakilas[1,#], Ihor Cherniukh[2,3,§], Kseniia Shcherbak[2,3], Chenglian Zhu[2,3], Maryna I. Bodnarchuk[2,3], Maksym V. Kovalenko[2,3], Rainer F. Mahrt[1], Gabriele Rainò[2,3], Thilo Stöferle[1,]*

[1] IBM Research Europe – Zurich, Säumerstrasse 4, 8803 Rüschlikon, Switzerland

[2] Department of Chemistry and Applied Biosciences, ETH Zurich, Vladimir Prelog Weg 1, 8093 Zürich, Switzerland

[3] Laboratory for Thin Films and Photovoltaics, Empa, Ueberlandstrasse 129, 8600 Dübendorf, Switzerland

* **Corresponding Author:** tof@zurich.ibm.com

# Present address: Paul Scherrer Institut, Forschungsstrasse 111, 5232 Villigen, Switzerland

§ Present address: Institute of Science and Technology Austria, Am Campus 1, 3400 Klosterneuburg, Austria

# QD	$\tau_{fast,out}$ (ps)	$\tau_{fast,in}$ (ps)	$F_P = \tau_{fast,out}/\tau_{fast,in}$	$\tau_{slow,out}$ (ps)	$\tau_{slow,in}$ (ps)	A_{in}/A_{out}
**1**	68 ± 1	28.1 ± 0.3	2.42 ± 0.04	258 ± 7	313 ± 7	2.8
**2**	153 ± 3	36.4 ± 0.4	4.20 ± 0.09	1060 ± 80	330 ± 6	5.3
**3**	111 ± 2	30.9 ± 0.4	3.59 ± 0.08	720 ± 42	278 ± 5	1.9
**4**	484 ± 3	39.1 ± 0.4	12.4 ± 0.1	-	278 ± 1	10
**5**	396 ± 4	42.5 ± 0.7	9.3 ± 0.2	-	278 ± 2	14.1
**6**	520 ± 3	28.7 ± 0.2	18.1 ± 0.2	-	255.4 ± 0.8	3.6

Table S1. Summary of the fitted decay times, Purcell factors and intensity ratios for the presented QDs. The decay traces for QD#1-3 (at 6 K), both inside and outside the cavity, are fitted using a double-exponential function with time constants τ_{fast} and τ_{slow}. For QD#4-6 (at 50 K), the traces inside the cavity are fitted with a double-exponential function, while those outside the cavity are fitted with a single-exponential function. The offset of the fitting functions is fixed to the average of the counts before the rising time. The Purcell factor, F_P, can be obtained from the ratio $\tau_{fast,out}$ / $\tau_{fast,in}$. The intensity ratio A_{in}/ A_{out} corresponds to the spectrally integrated emission intensity enhancement inside versus outside the cavity.

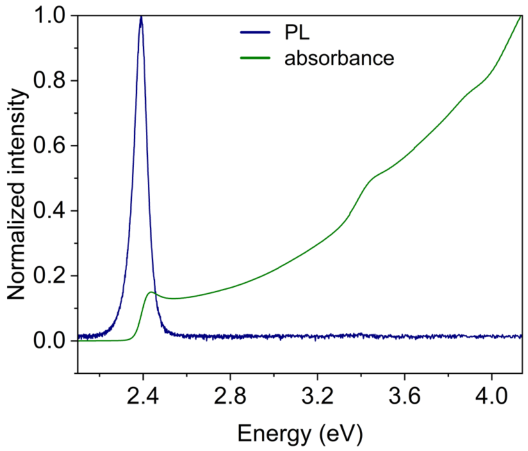

Figure S1. PL and absorption spectra of $CsPbBr_3$ QDs. Normalized PL (blue curve) and absorption (green curve) spectrum of 25 nm sized QDs dispersed in solution and measured at room temperature**.**

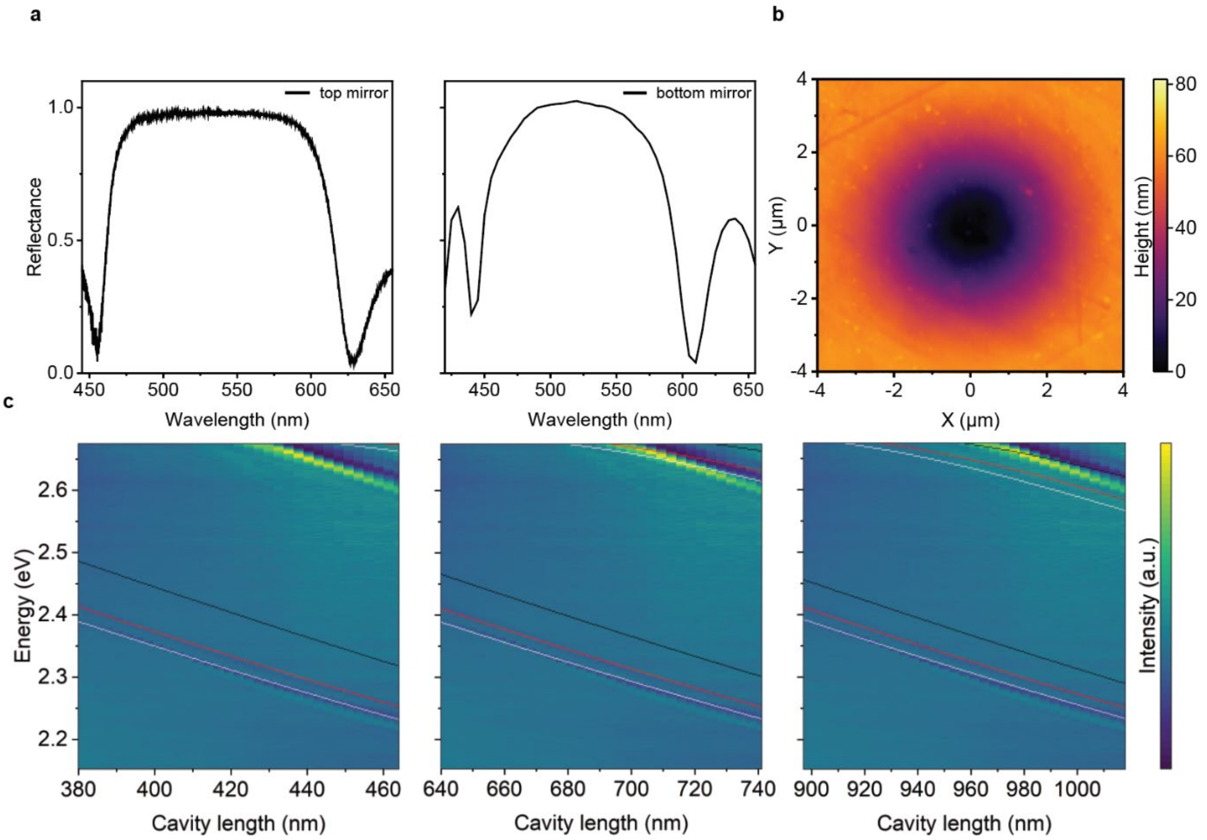

Figure S2. Cavity characterization. (a) On the left, experimental reflectance spectrum of the top DBR mirror, measured at normal incidence with a white-light reflection measurement. On the right, experimental reflectance spectrum of the bottom DBR mirror, measured at an incidence angle of 20° with a spectroscopic ellipsometer. (b) Atomic force microscopy image of the Gaussian-shaped deformation, showing a FWHM of ~4 µm and a depth of ~60 nm. (c) Comparison between the white-light reflection measurement (colormap) and transfer-matrix simulations for planar modes of different longitudinal order (black lines) to extract the correct range of cavity length. To account for the Gaussian potential, each planar mode of different longitudinal order is horizontally shifted with two different offsets to overlap with both the LG_{00} and LG_{01} modes. These shifted planar modes are displayed as white and red lines, respectively. The shifts for the LG_{00} and LG_{01} are taken from the eigenvalues obtained by solving the 2D Schrödinger equation, assuming a Gaussian potential with a spatial depth of 60 nm and a FWHM of 4 µm. The spatial depth is converted into energy by evaluating the energy shift of a planar mode when the cavity length is varied by 60 nm. Since the slope of a planar mode depends on the longitudinal order and, therefore, on the cavity length, the potential depth varies across the three panels: 115 meV (left), 94 meV (centre) and 80 meV (right). The resulting eigenvalues are divided by the corresponding potential depth and scaled by 60 nm to retrieve the spatial shifts.

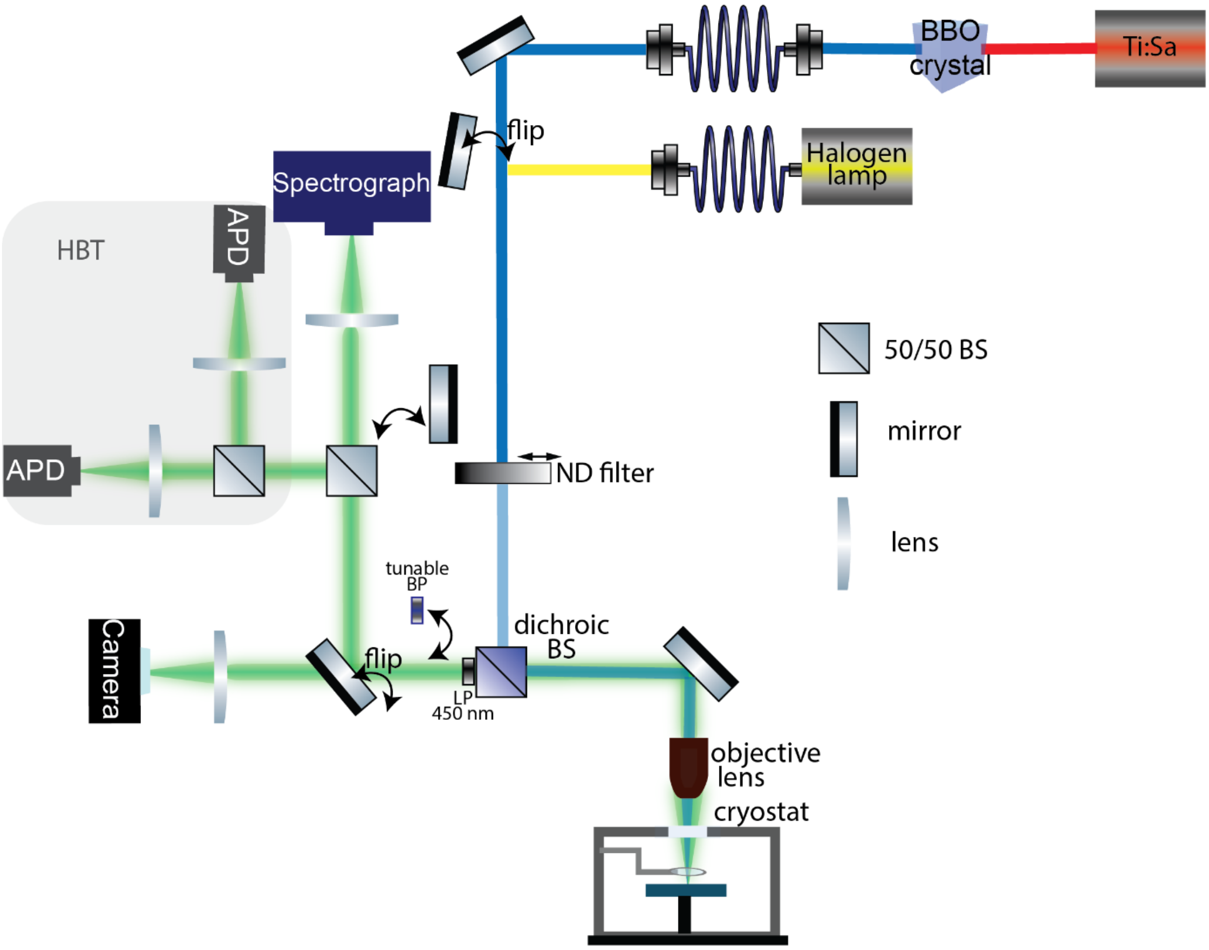

Figure S3. Optical setup. Schematic of the experimental setup where both cavity halves are mounted on XYZ nanopositioners inside a liquid Helium flow cryostat.

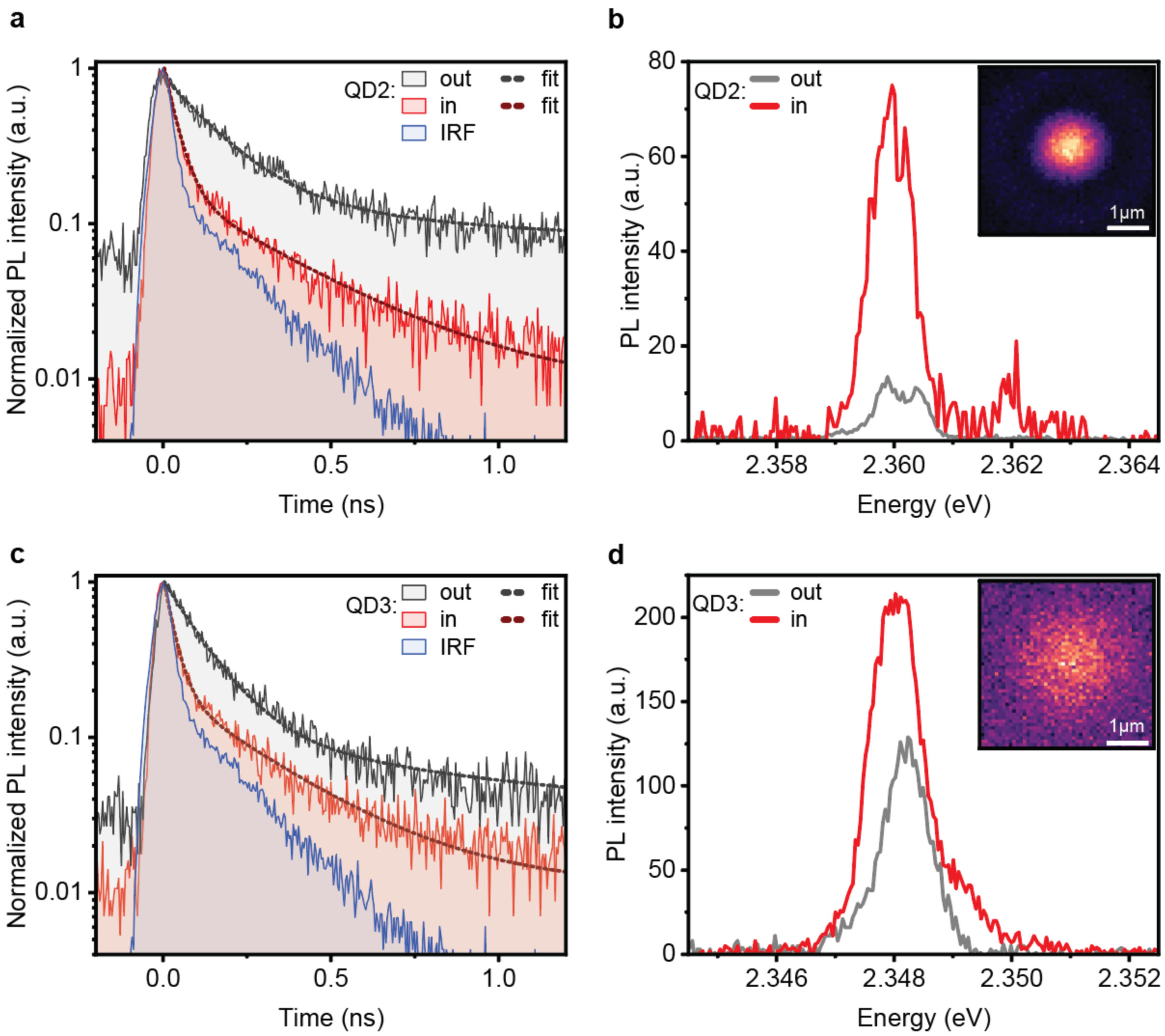

Figure S4. Optical study of QD#2, 3 at 6 K. Decay traces (a),(c) and PL spectrum (b),(d) of QD#2 (a),(b) and QD#3 (c),(d) placed inside (red) and outside (gray) the cavity, with the IRF (blue) as reference. The insets in (b),(d) show the respective measured real-space mode profile.

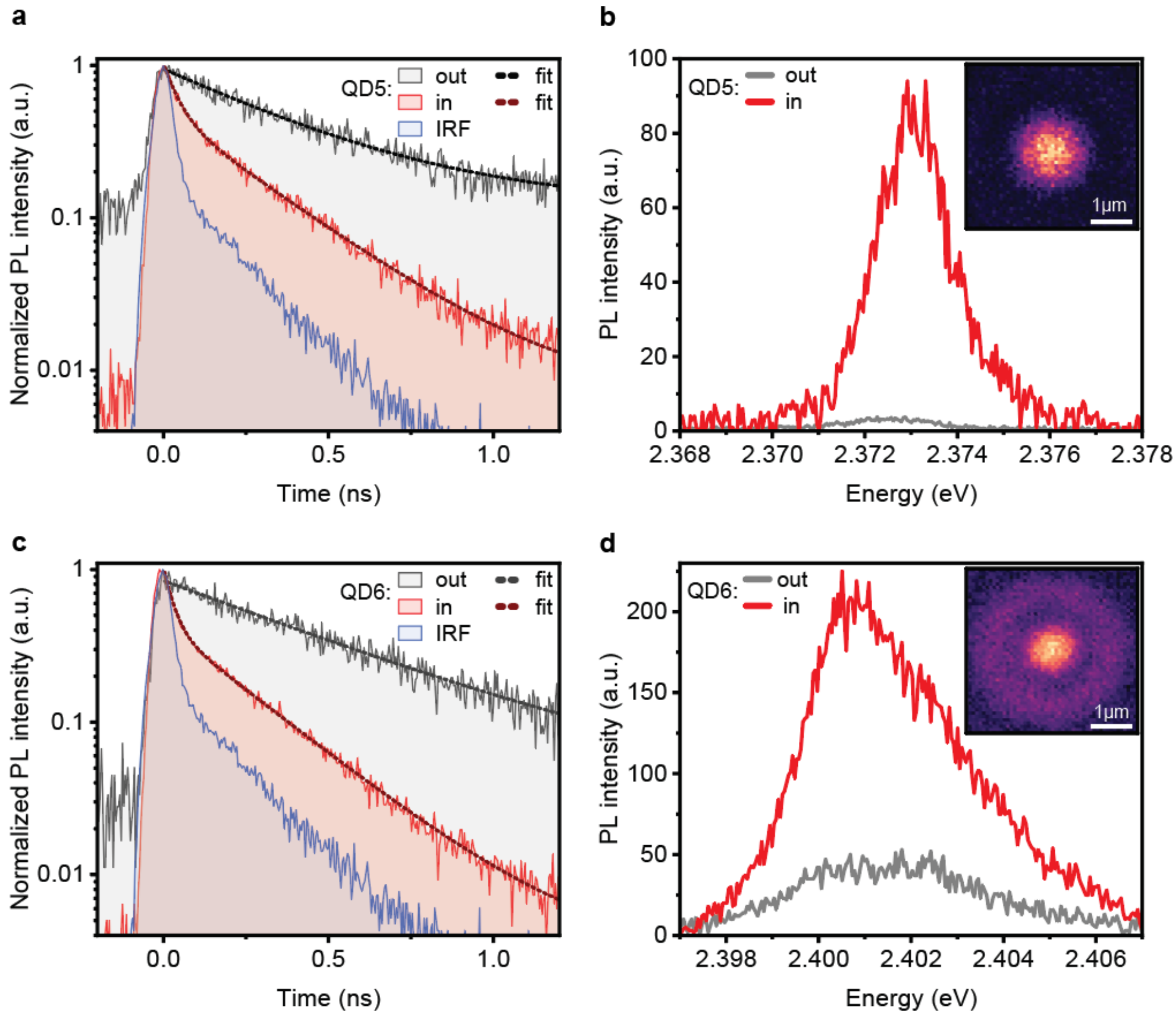

Figure S5. Optical study of QD#5, 6 at 50 K. Decay traces (a),(c) and PL spectrum (b),(d) of QD#5 (a),(b) and QD#6 (c),(d) placed inside (red) and outside (gray) the cavity, with the IRF (blue) as reference. The insets in (b),(d) show the respective measured real-space mode profile.

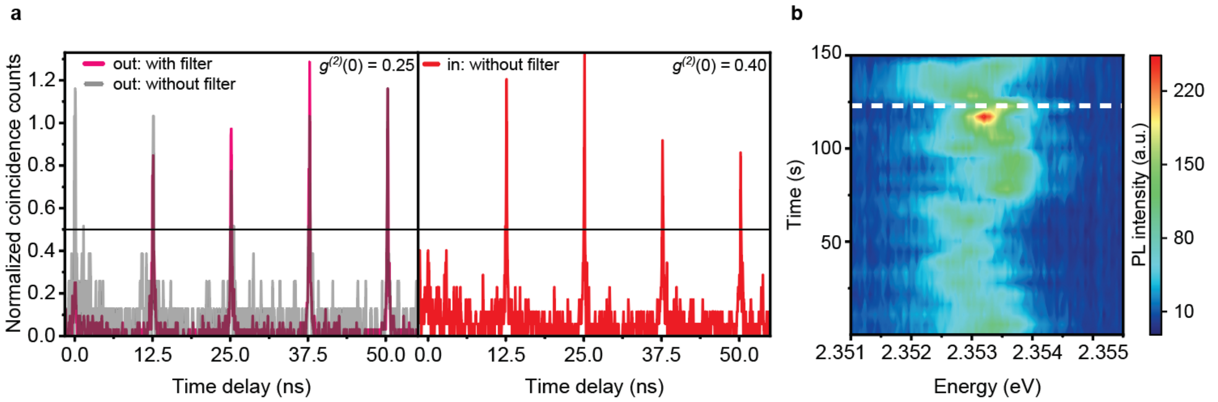

Figure S6. $g^{(2)}(0)$ and time-series measurements for QD#1. (a) $g^{(2)}(0)$ measurements outside (left panel) and inside (right panel) the cavity. A $g^{(2)}(0)$ value of 0.25 is obtained when an additional bandpass filter is used (pink line) while a $g^{(2)}(0)$ close to 1 is measured when the bandpass filter is removed (gray line). Inside the cavity, a $g^{(2)}(0)$ value of 0.4 is achieved without the use of any filter. These values are extracted from the peak maxima of the raw, uncorrected data and the normalization is retrieved by dividing the coincidence counts by the average of the peak maxima at time delays different from 0. (b) PL time-series (outside the cavity), acquired with an integration time of 5 s. The dashed white line highlights the time at which the spectrum is shown in Figure 2b.

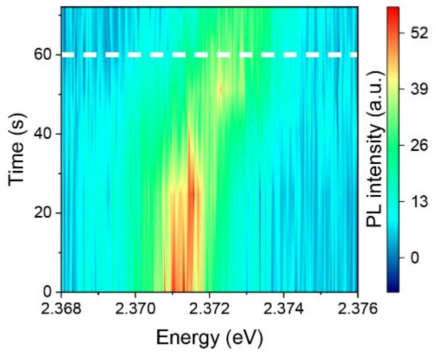

Figure S7. Time-series measurements for QD#5. PL time-series of QD#5. The measurement is acquired when the QD is outside the cavity with an integration time of 10 s. The dashed white line indicates the time at which the spectrum is shown in Figure S5b.

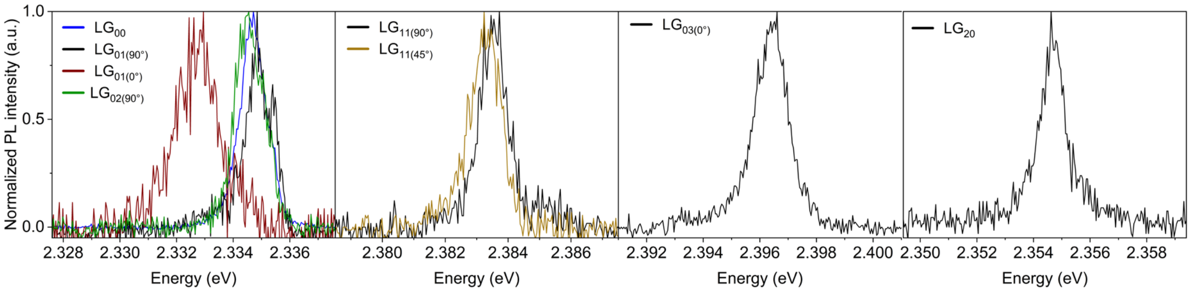

Figure S8. Spectra of the LG$_{nl}$ modes in Figure 3. Normalized PL intensity as a function of energy of the LG$_{nl}$ modes shown in Figure 3. The panels are grouped for the same single QDs inside the microcavity, when the LG modes are changed by in-situ tuning.
